# Community-Driven, Text Message–Based COVID-19 Surveillance System, Los Angeles County, California, USA, 2020–2024

**DOI:** 10.3201/eid3111.250907

**Published:** 2025-11

**Authors:** Jordan B. Braunfeld, Elizabeth Traub, Howard Chiou, Aryana T. Amoon, Christina Collins, Allison Joyce, Justin Buendia, Prabhu Gounder, Annabelle de St. Maurice

**Affiliations:** Centers for Disease Control and Prevention, Atlanta, Georgia, USA (J.B. Braunfeld); Los Angeles County Department of Public Health, Los Angeles, California, USA (J.B. Braunfeld, E. Traub, H. Chiou, A.T. Amoon, C. Collins, A. Joyce, J. Buendia, P. Gounder, A. de St. Maurice)

**Keywords:** COVID-19, Surveillance, respiratory infections, viruses, SARS-CoV-2, influenza, California, United States

## Abstract

Respiratory virus indicators were unreliable at the onset of the COVID-19 pandemic when testing availability was limited and residents with mild symptoms were advised to avoid unnecessary medical care. The Los Angeles County Department of Public Health (Los Angeles, California, USA) developed Angelenos in Action (AiA), a text message–based community syndromic surveillance system to monitor respiratory illness trends. Approximately 17,000 unique participants responded >1 time; 43% of participants continue to regularly respond to the survey. We assessed AiA’s performance by measuring correlation coefficients with reported COVID-19 case counts (0.975), sentinel laboratory SARS-CoV-2 test positivity rate (0.762), and wastewater SARS-CoV-2 concentrations (0.861). AiA performed strongly against 3 comparator surveillance methods and correlated particularly well with raw case counts. A moderate correlation was also noted between influenza test positivity rate and AiA data, indicating the system has potential to detect respiratory illness besides COVID-19.

Even before the COVID-19 pandemic, a primary challenge with respiratory disease surveillance has been estimating the number of infections within the community, because counting every case of illness was not feasible. Public health officials have mitigated this limitation by using proxy indicators to detect rises in community incidence of disease. Commonly used proxies include sentinel laboratory data, which examine the percentage of respiratory viral tests returning positive, and syndromic surveillance data, which tracks the percentage of emergency department (ED) visits for respiratory viral illness. As such, measuring the activity of respiratory diseases generally relies on ill persons seeking medical care and getting tested.

That approach to viral respiratory disease surveillance was disrupted during the COVID-19 pandemic. Early during the pandemic, to limit effects on the healthcare system and decrease individual risk, the public was asked to stay home and only seek care if severely ill (*1*). As a result, ED visit volume declined precipitously (*2*). In addition, SARS-CoV-2 testing availability was limited, and its use was often restricted to patients deemed sick enough to require medical care (*3*). The Los Angeles County (LAC) Department of Public Health (DPH), in Los Angeles, California, USA, identified the need to develop a system to gather timely, accurate, and relevant information about SARS-CoV-2 circulation in the community. In response, Angelenos in Action (AiA), a text message-based community syndromic surveillance system to track respiratory symptom trends, was created. AiA was modeled after long-running community surveys, such as Influenzanet and Outbreaks Near Me (*4*–*7*), and was intended to track trends in respiratory viral illness by measuring the number of persons reporting respiratory viral symptoms. The survey was intentionally focused on symptom reporting as a means of capturing illnesses of varying severity, including those cases mild enough to not require a medical visit.

For ≈4 years, AiA has provided data for LAC DPH. However, we wished to perform a comprehensive longitudinal analysis to ascertain whether it succeeded in reliably tracking communitywide COVID-19 activity. Our aim is to assess the performance of AiA as a method of tracking COVID-like illness (CLI) in LAC by observing its timeliness and accuracy in correlation to other measures of COVID incidence.

## Materials and Methods

The symptom survey became available to all LAC residents >18 years of age on July 6, 2020. Recruitment for survey participants was performed with the intent to create a respondent panel that was demographically similar to the overall LAC population. Participant recruitment occurred during late summer and late fall of 2020. Participants were recruited primarily through digital advertising and social media advertising on Facebook (http://facebook.com). Two local radio stations recorded advertising spots that aired Monday through Wednesday for the first 2 weeks of the launch. Their websites and social media accounts displayed digital banners and posts about AiA during July 2020. Advertisements also ran on 2 local African American–focused digital newspapers. DPH generated press releases in both English and Spanish and advertised AiA through DPH social media accounts. An external marketing agency managed paid media. Recruitment materials were approved by the LAC DPH institutional review board. Enrollment has remained continuously open to all LAC residents. Project participation was initially limited only to those with a cell phone; however, an option to participate by email was added on December 20, 2021. Surveys were generated by Qualtrics (https://www.qualtrics.com) and sent by short message service. At the time of enrollment, participants were offered a choice of receiving the survey in English or Spanish. Participants provided their postal (ZIP) code to confirm residence in LAC and either a telephone number or an email address. Additional questions about age, sex, and race and ethnicity were optional. Participants may unsubscribe at any time by replying STOP to any AiA text message.

**Figure 1:**
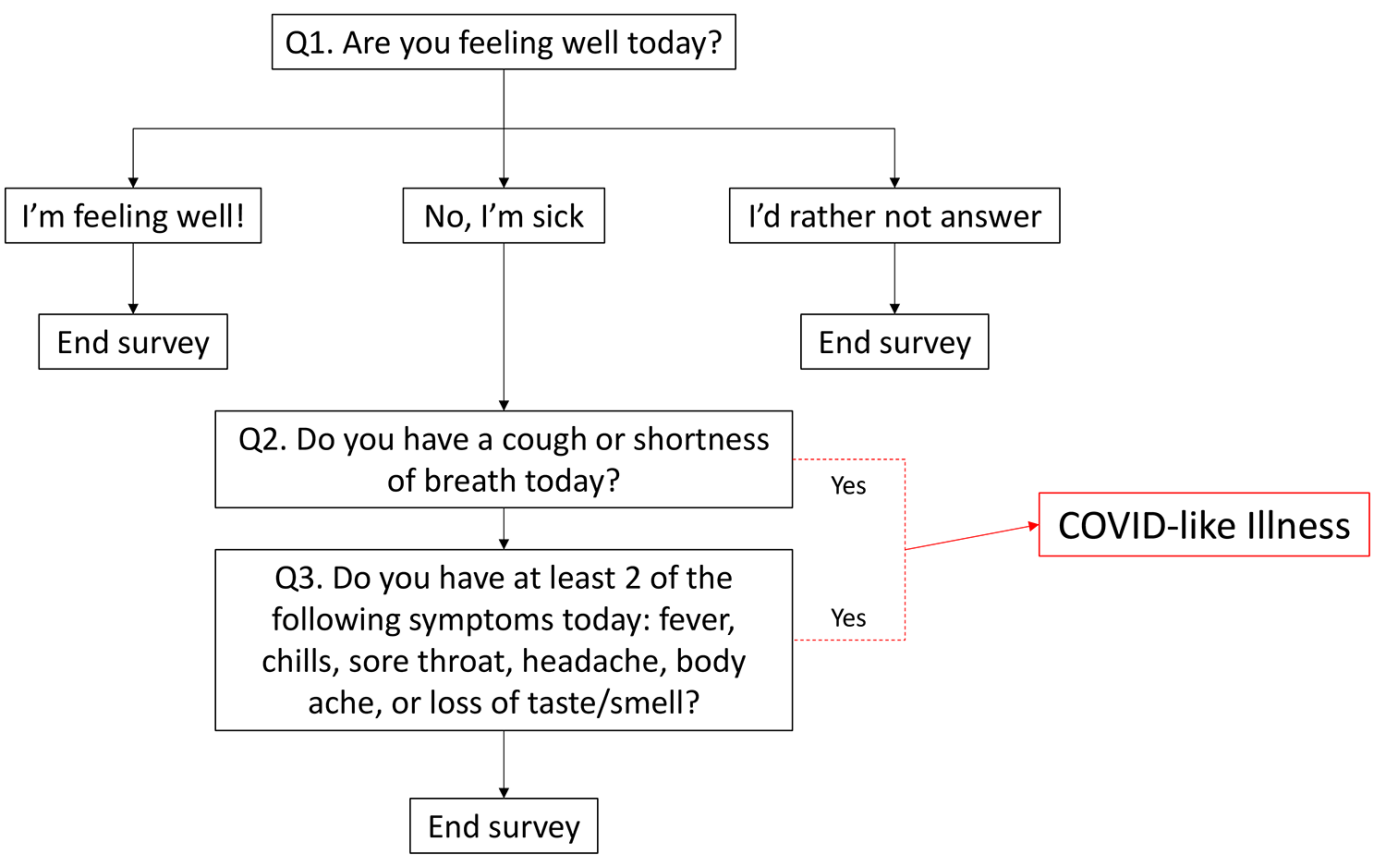
Survey question and response flow for the Angelenos in Action community-driven, text message-based COVID-19 surveillance system used in Los Angeles County, California, USA, 2020–2024. Red represents response pattern that results in the designation of COVID-like illness.

The symptom survey was distributed weekly and consisted of 1 question asking the respondent whether they were feeling well that day. If they reported not feeling well, 2 additional yes or no questions were asked. Participants were asked whether they were experiencing cough or shortness of breath and whether they were experiencing >2 of the following symptoms: headache, body ache, sore throat, fever, chills, or loss of sense of taste or smell (Figure 1). In consideration of the Council of State and Territorial Epidemiologists list of COVID-19 clinical symptoms at the time of the survey’s development (*8*), respondents were considered to have CLI if they responded yes to both questions. Respondents who reported not feeling well, regardless of their responses to the symptom-specific questions, were instructed to contact their doctor.

Active respondents for each year were defined as those who submitted >1 survey during that Morbidity and Mortality Weekly Report (MMWR) surveillance year (MMWR week 40 of that calendar year through MMWR week 39 of the next calendar year) (*9*). To observe survey response trends over time, responses were tracked by MMWR week and plotted by MMWR surveillance year. To account for the 53rd week during the 2020–21 surveillance year, 2020 week 40 is an imputed average of the CLI rates during week 40 and week 41. The data for each remaining week during 2020 were subsequently shifted 1 week backward. We chose to impute an average for week 40 and week 41 because there is historically little variation in respiratory viral activity during those weeks (*10*). We assessed survey population representativeness by comparing survey participant demographics with LAC demographic characteristics derived from the provisional population estimates prepared by Hedderson Demographic Services for the LAC Internal Services Department (*11*).

We compared trends in AiA’s CLI rate with data from 3 other methods of COVID-19 surveillance used by LAC DPH during the pandemic; raw COVID-19 case counts, sentinel laboratory data, and wastewater surveillance data. We tabulated raw countywide case counts by mandatory laboratory and provider case reports to LAC; counts are described as 7-day rolling averages (*12*). We described sentinel laboratory data as weekly aggregates of the percentage of positive tests out of all tests performed for a given virus at the 7 LAC sentinel laboratories (*10*). We gathered sentinel laboratory data for SARS-CoV-2, influenza, and respiratory syncytial virus (RSV) testing. Wastewater surveillance for SARS-CoV-2 has been ongoing in LAC since January 9, 2022, and has involved all 3 LAC water treatment plants since September 12, 2022. Together, the 3 plants service ≈7.7 million of LAC’s almost 10 million inhabitants. Each plant collects an average of 3 samples per week. We gathered wastewater surveillance data from the WastewaterSCAN dashboard (*13*,*14*). We describe wastewater SARS-CoV-2 RNA concentrations as a smoothed average of 5 consecutive samples after trimming the highest and lowest of the 5 samples.

We calculated CLI rates as the proportion of all responses in the AiA survey that fulfilled criteria for CLI. We calculated Pearson correlation coefficients to assess linear correlation between CLI rates and each of the 3 comparator metrics. The time frame during which CLI rates were calculated differed depending on the comparison being made. We reported CLI rates as 7-day rolling averages in comparison with daily case counts, weekly averages in comparison with sentinel laboratory data, and 7-day smoothed averages in comparison with wastewater surveillance data. Because symptoms of CLI overlap with similar respiratory illnesses, including influenza and RSV, Pearson correlation coefficients were also calculated between CLI rates and weekly LAC sentinel laboratory test positivity rates for influenza and RSV.

Because of prominent shifts in testing strategies and major changes to laboratory and provider reporting requirements in LAC throughout 2022, we calculated correlations between CLI rates and reported cases by using data from the opening of the survey through January 29, 2022. We calculated correlations between CLI rates and sentinel laboratory data for SARS-CoV-2 by using data from the opening of the survey through April 6, 2024. Because influenza viruses and RSV were essentially not circulating during the first year of the pandemic, we calculated supplemental correlations between CLI rates and sentinel laboratory data for influenza and RSV by using data collected during June 20, 2021–April 6, 2024. We calculated correlations between CLI rates and wastewater data by using data collected during January 9, 2022–April 6, 2024.

To determine if one system’s data lagged another system, we calculated cross-correlation for each comparison by offsetting the data from 1 system by a discrete time interval and obtaining a new Pearson correlation coefficient. We repeated this process by shifting data 5 intervals forward and backward. The time interval used in the comparisons to case counts and to wastewater data was 1 day. The time interval used in the comparison to sentinel laboratory data was 1 week. If the offset data resulted in a higher Pearson correlation coefficient than in the original comparison, we determined there was a data lag. 

This work was reviewed by Centers for Disease Control and Prevention and the LAC DPH Institutional Review Boards, was not considered human subjects research, and was conducted consistent with applicable federal law and Centers for Disease Control and Prevention policy. Consent from survey participants was obtained at the time of enrollment.

## Results

### AiA Participation

**Figure 2:**
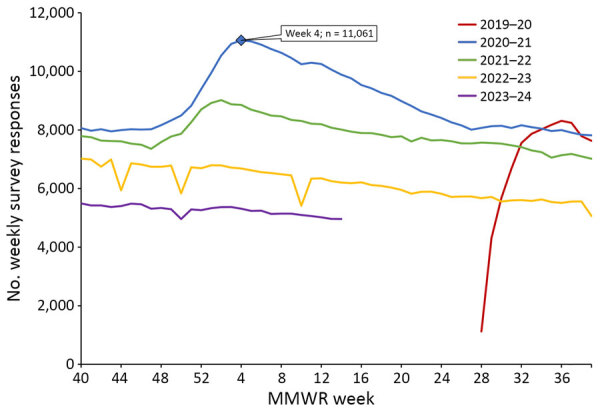
Trends in the weekly number of responses to the Angelenos in Action community-driven, text message–based COVID-19 surveillance system by MMWR influenza season (*10*), Los Angeles County, California, USA, 2020–2024. The blue diamond represents the highest number of surveys submitted in any week since the survey opened. MMWR, Morbidity and Mortality Weekly Report.

During July 6, 2020–April 6, 2024, a total of 20,033 unique adults received >1 symptom survey from AiA. The highest number of participants receiving a symptom survey in a week was 17,393 during the week starting December 26, 2021. The highest number of survey responses in 1 week was 11,061 during the week starting January 24, 2021 (Figure 2). There were 7,792 responses during the first week of the 2021–22 season, 7,022 responses during the first week of the 2022–23 season, and 5,493 responses during the first week of the 2023–24 season. The survey has received >1 response from 17,092 unique adults (Table). Respondents identifying as White composed 49.9% of all survey respondents and 51.3% of active respondents during the 2023–24 season, whereas 29.2% of all LAC residents identify as White. Respondents identifying as Latino composed 29.2% of all survey respondents and 28.0% of active respondents during the 2023–24 season, whereas 45.9% of all LAC residents identify as Latino. Respondents >40 years of age composed 68.9% of all survey respondents and 78.3% of active respondents during the 2023–24 season, whereas 60.6% of all LAC residents are >40 years of age. Active respondents declined each year from a peak of 14,587 during the 2020–21 season to 7,388 during the 2023–24 season through the week ending April 6, 2024. When excluding the truncated 2019–20 and 2023–24 seasons, the average number of surveys submitted by respondents completing the survey >1 time in a year increased from 32.5 during the 2020–21 season to 33.9 during the 2022–23 season. (Appendix Table).

**Table:** Demographic characteristics of Los Angeles County adults and respondents to the Angelenos in Action community-driven, text message–based COVID-19 surveillance system, California, USA, 2020–2024*

Characteristics	No. (%)		
	Los Angeles County adult population	Respondents who answered >1 survey†	Active respondents‡ during 2023–2024 season†
Total	7,364,388	17,092	7,388
Age, y			
18–29	1,540,122 (20.9)	1,388 (8.1)	328 (4.4)
30–39	1,360,790 (18.5)	3,881 (22.7)	1,256 (17.0)
40–49	1,260,968 (17.1)	4,751 (27.8)	2,033 (27.5)
50–59	1,251,487 (17)	3,813 (22.3)	1,967 (26.6)
60–69	1,058,203 (14.4)	2,396 (14)	1,345 (18.2)
>70	892,818 (12.1)	814 (4.8)	440 (6)
Not given	NA	49 (0.3)	19 (0.3)
Sex			
M	3,601,392 (48.9)	3,995 (23.4)	1,640 (22.2)
F	3,762,996 (51.1)	12,984 (76)	5,708 (77.3)
Not given	NA	70 (0.4)	23 (0.3)
Race			
American Indian	14,582 (0.2)	51 (0.3)	23 (0.3)
Asian	1,218,430 (16.5)	1,668 (9.8)	693 (9.4)
Black	590,152 (8)	493 (2.9)	250 (3.4)
Hawaiian/Pacific Islander	13,467 (0.2)	77 (0.5)	33 (0.4)
Latino	3,380,602 (45.9)	4,985 (29.2)	2,068 (28.0)
Multiracial	NA	845 (4.9)	379 (5.1)
White	2,147,155 (29.2)	8,530 (49.9)	3,792 (51.3)
Other	NA	238 (1.4)	77 (1)
Not given	NA	205 (1.2)	73 (1)

*NA, not available. †Through April 6, 2024. ‡Active respondents are defined as those who submitted >1 survey during that year’s Morbidity and Mortality Weekly Report influenza season (MMWR week 40 of that year through MMWR week 39 of the next year) (10).

### CLI Detection and System Comparisons

**Figure 3:**
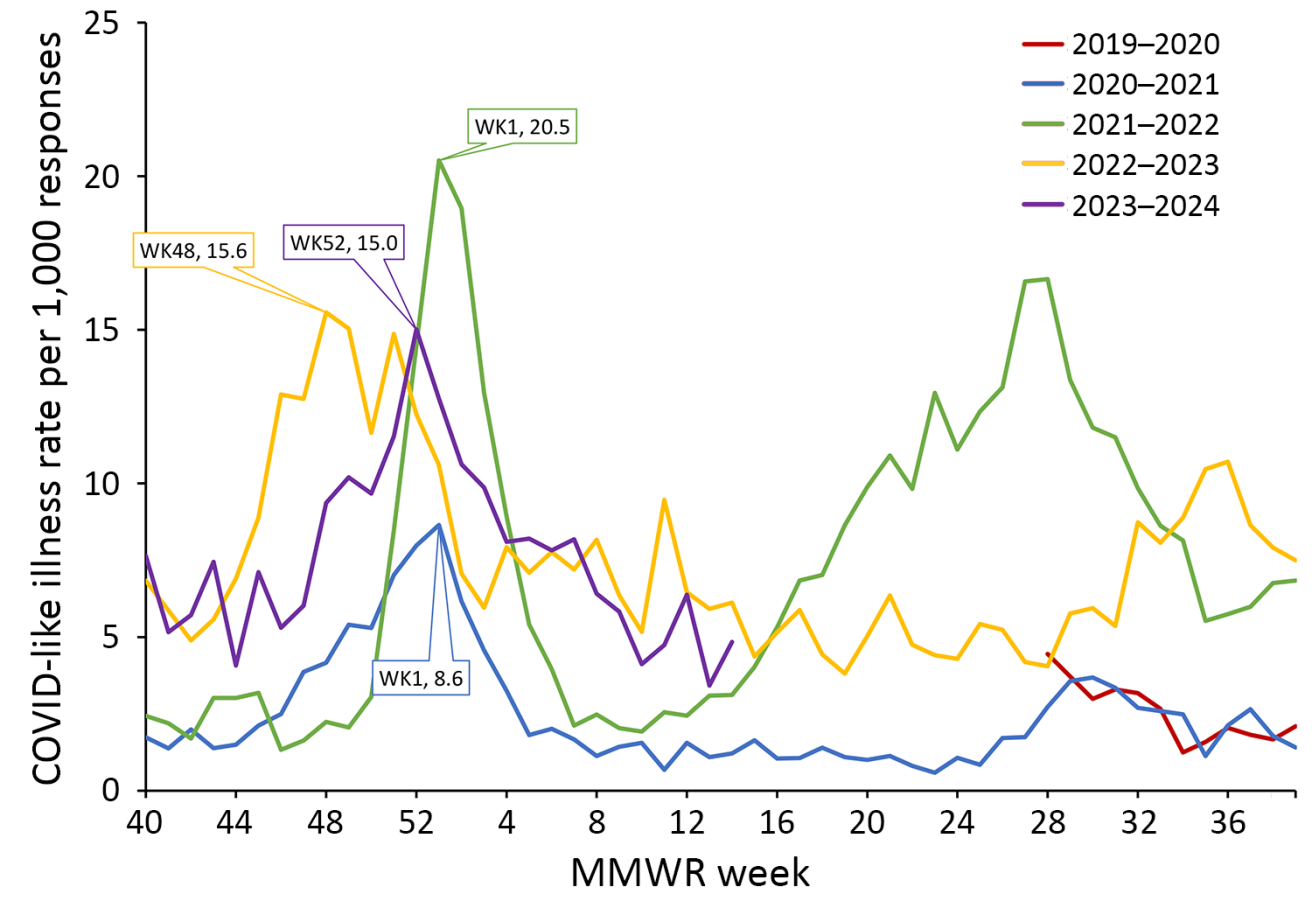
Trends in COVID-like illness responses to the Angelenos in Action community-driven, text message–based COVID-19 surveillance system used in Los Angeles County, California, USA, 2020–2024, by MMWR influenza season (*10*). A response was designated as a COVID-like illness if the respondent indicated they were sick and answered yes to both symptom-specific questions. The colored diamonds correspond to the peak rate for each year. MMWR, Morbidity and Mortality Weekly Report; WK, week.

**Figure 4:**
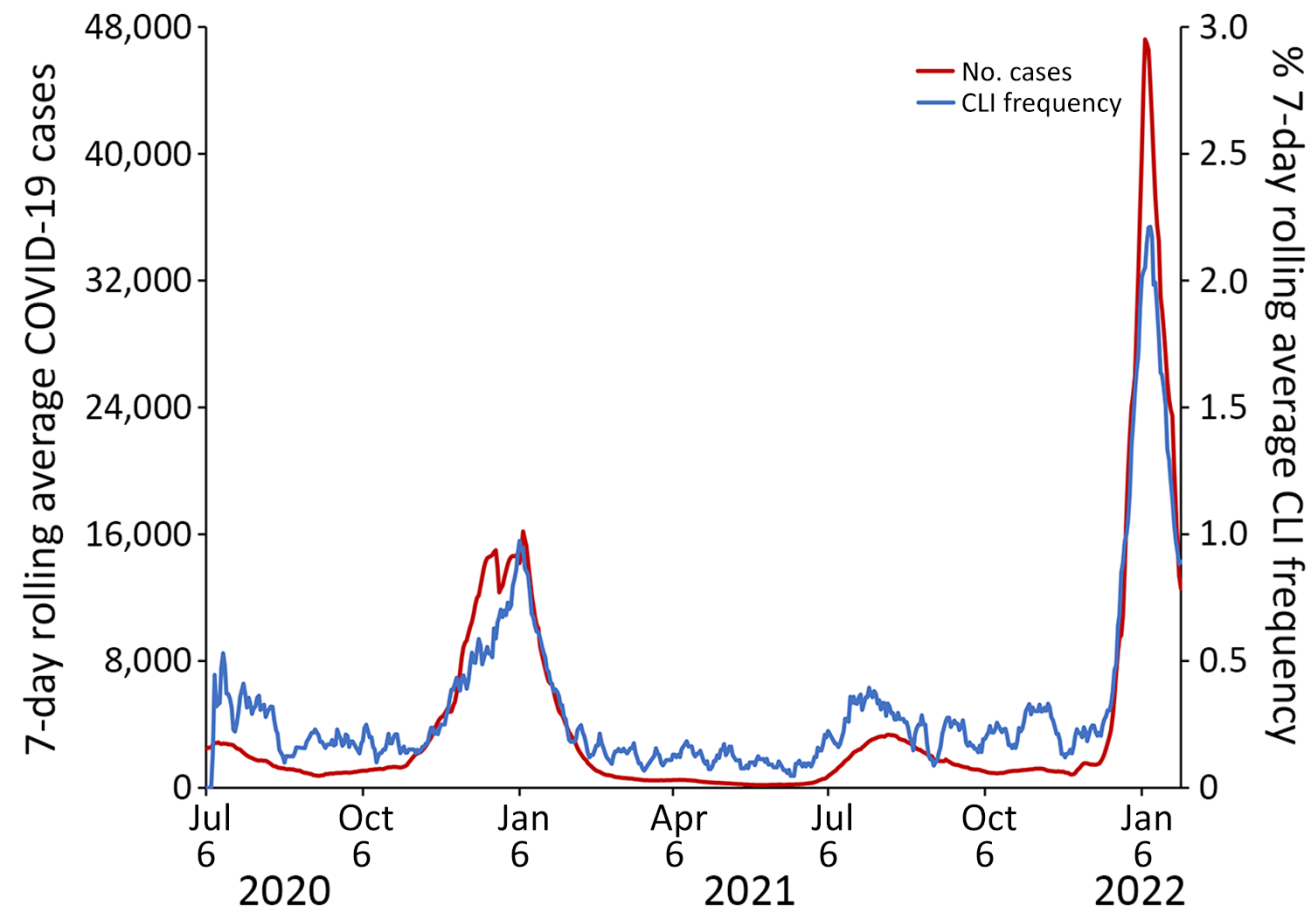
Trends of 7-day rolling average CLI response rate to the Angelenos in Action community-driven, text message–based COVID-19 surveillance system used in Los Angeles County, California, USA, 2020–2024, and 7-day rolling average Los Angeles County COVID-19 case counts during July 6, 2020–January 29, 2022. A response was designated as a CLI if the respondent indicated they were sick and answered yes to both symptom-specific questions. Pearson correlation coefficient = 0.975. CLI, COVID-like illness.

**Figure 5:**
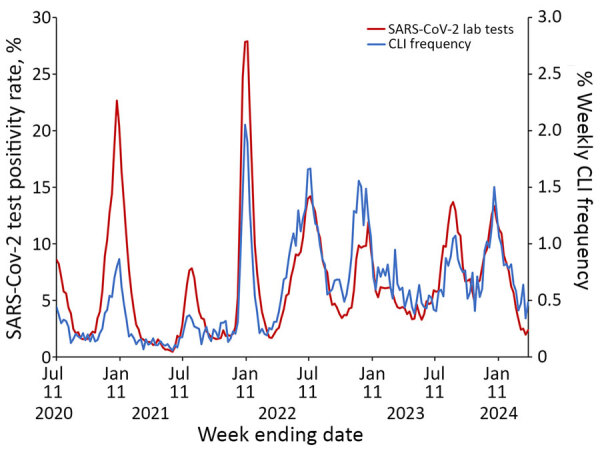
Trends of weekly CLI response rate to the Angelenos in Action community-driven, text message–based COVID-19 surveillance system used in Los Angeles County, California, USA, 2020–2024, and weekly Los Angeles County sentinel laboratory SARS-CoV-2 percentage positivity during July 6, 2020–April 6, 2024. A response was designated as a CLI if the respondent indicated they were sick and answered yes to both symptom-specific questions. Pearson correlation coefficient = 0.762. Scales for the y-axes differ substantially to underscore patterns but do not permit direct comparisons. CLI, COVID-like illness.

**Figure 6:**
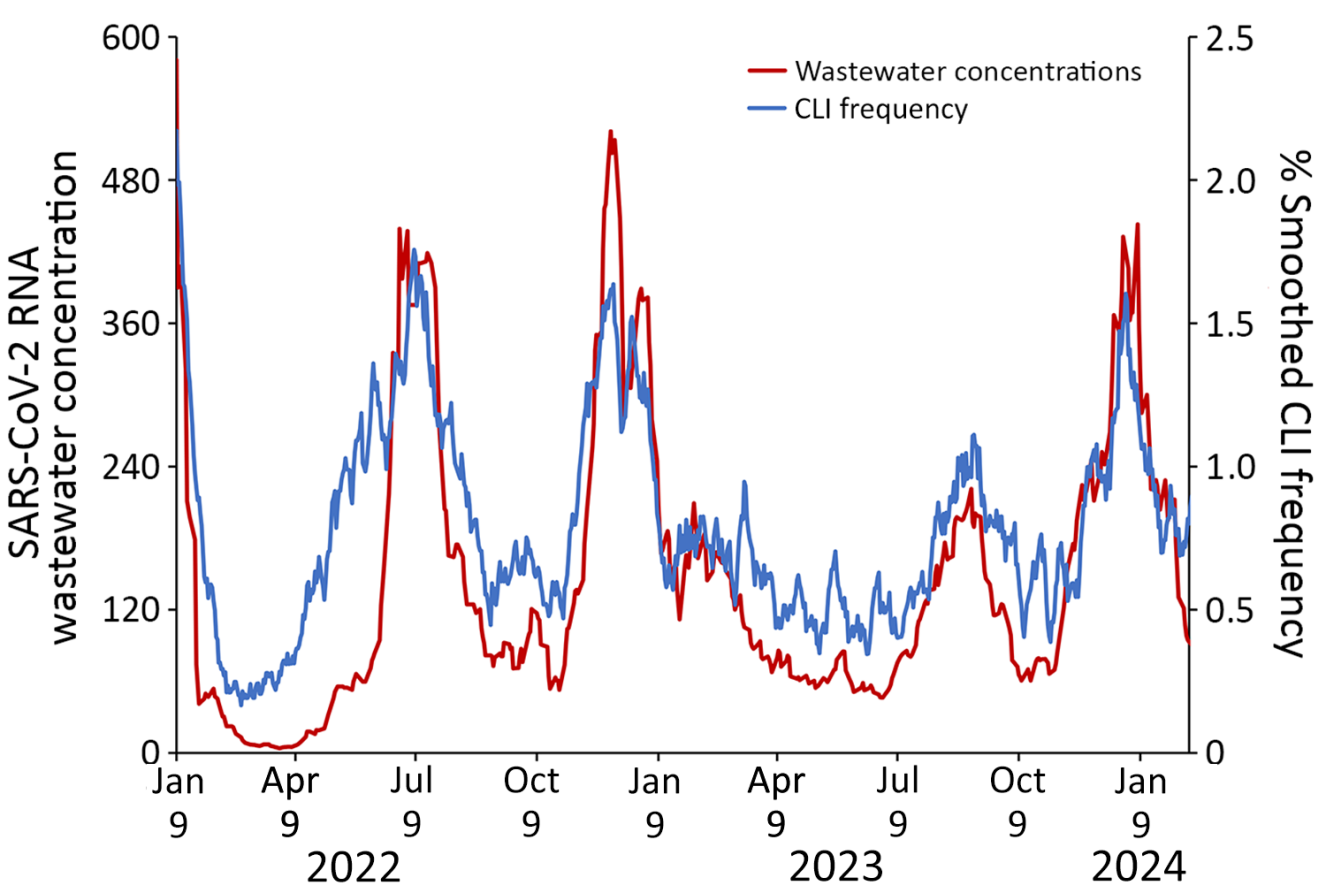
Trends of smoothed CLI response rate to the Angelenos in Action community-driven, text message–based COVID-19 surveillance system used in Los Angeles County, California, USA, 2020–2024, and trimmed and smoothed Los Angeles County SARS-CoV-2 RNA wastewater concentrations during January 9, 2022–April 6, 2024. A response was designated as a CLI if the respondent indicated they were sick and answered yes to both symptom-specific questions. Concentrations are normalized against values of a ubiquitous virus to account for stool flow rates and then multiplied by a factor of 10^6^ (data not shown). Pearson correlation coefficient = 0.861. CLI, COVID-like illness.

CLI rate increased each season from 2.2/1,000 responses during the 2019–20 season to 7.6/1,000 responses during the 2023–24 season (Appendix Table). CLI rate peaked during MMWR week 1 during both the 2020–21 season and the 2021–22 season (Figure 3). CLI rate peaked during MMWR week 48 during the 2022–23 season and MMWR week 52 during the 2023–24 season. When comparing AiA CLI rates to reported COVID-19 case count data, the Pearson correlation coefficient was 0.975 (Figure 4). When comparing AiA CLI rates to sentinel laboratory percent positivity data for SARS-CoV-2, the Pearson correlation coefficient was 0.762 (Figure 5). When comparing AiA CLI rates to wastewater surveillance data for SARS-CoV-2, the Pearson correlation coefficient was 0.861 (Figure 6). An increase in CLI rate was noted during May and June 2022 when no similar rise in wastewater detection of SARS-CoV-2 RNA was reported. Cross-correlation did not identify a data lag in any of those 3 comparisons. Additional comparisons of AiA CLI rates to sentinel laboratory percentage positivity data for RSV (Appendix Figure 1) produced a correlation coefficient of 0.094 and for influenza (Appendix Figure 2) produced a correlation coefficient of 0.508.

## Discussion

During the first 4 years of the COVID-19 pandemic, AiA was able to reliably track population-level trends in symptomatic respiratory illness without a lag in data signal when compared with other commonly used surveillance methods. Data from AiA correlated well with data from the 3 comparator systems; the lowest correlation existed between AiA data and SARS-CoV-2 sentinel laboratory percentage positivity data. We noted multiple factors that might have contributed to the relatively weaker correlation. First, sentinel laboratory data was delivered in the form of once-weekly 7-day averages, so this comparison included fewer data points than either of the other 2 comparisons. Second, visual inspection of the data in this comparison shows that CLI increases from AiA data might have occasionally preceded percentage positivity increases from sentinel laboratory data. This effect was particularly apparent during the emergence of the highly infectious Delta variant during summer 2021 and during the winter 2022 seasonal increase. Last, although cross-correlation reassuringly displayed no overall lag in either dataset, this method might also be limited by the spacing of data points (i.e., a lag of <7 days might exist but would not be possible to detect by this method).

When other respiratory viruses resumed circulating at higher levels in late 2021, data from AiA continued to correlate well with COVID-19 data from other systems. However, AiA’s CLI trends also appeared to have been affected by >1 other viruses. Whereas AiA showed a weak correlation with RSV sentinel laboratory data (Appendix Figure 1), it showed a moderate correlation with influenza sentinel laboratory data (Appendix Figure 2). When examining periods of increased viral activity specifically, we noted that an instance of unseasonably high influenza activity during May and June 2022 appeared to correspond to a rise in CLI that preceded the increase in SARS-CoV-2 test positivity rates and SARS-CoV-2 wastewater concentrations observed later that summer. Further, when compared with peak SARS-CoV-2 test positivity rates, peak CLI rates appeared proportionally higher when influenza and SARS-CoV-2 were co-circulating (July 2022 and December 2022, and January and December 2023) than when SARS-CoV-2 was circulating alone (July 2021, January 2022, and August and September 2023). Those findings indicates that AiA data might have been meaningfully influenced by influenza virus circulation, which is not surprising because of the overlap between typical influenza symptoms and the symptoms captured in the AiA symptom survey and highlights a limitation of the short survey format (*15*). This lower specificity means data from AiA must be interpreted in the context of other surveillance data and viral seasonality. However, it also demonstrated that AiA possesses potential to be used to detect respiratory illnesses beyond just COVID-19.

The simple data flow within AiA avoids the reporting delays inherent to the surveillance methods used before the pandemic. For example, electronic laboratory reporting data can take 3–4 days from specimen collection to test reporting. Syndromic surveillance requires appropriate medical record coding before reporting to public health, a process that can take 1–2 weeks. Because those systems rely on engagement with healthcare to capture cases, they are subject to additional delays attributable to the time from a patient first experiencing symptoms to their engagement with healthcare (*4*,*16*,*17*). For wastewater surveillance, the time required to collect and transport wastewater samples for testing introduces delays as long as 1–2 weeks. However, AiA delivers data with a remarkably short turnaround time, in as few as 24 hours after survey response. AiA’s data collection is also independent of healthcare seeking, so it is not subject to delays from symptom onset to a medical visit. This attribute also enables AiA to capture complete data on those who do not seek medical care at all, which proved to be another strength, because most viral respiratory illnesses, COVID-19 included, do not prompt healthcare seeking (*18*–*20*). As a result, AiA’s ability to rapidly display changes in disease activity and capture data regarding illnesses that did not require medical care made it particularly useful to DPH when the community health landscape was often rapidly changing.

LAC residents continue to participate in AiA >4 years after the pandemic started. Approximately 40% of all participants who responded since the survey’s inception have participated in >1 of the weekly surveys during the 2023–24 season. Further, the average number of responses submitted by each active respondent has been consistently high and increased in each full season of the analysis, indicating that those who continued to engage with the survey did so with consistently high frequency (Appendix Table 1). This result might be partially attributable to AiA’s text-based format and maximum of 3 questions per week, which placed minimal participation burden on respondents. This robust level of engagement was unexpected because no formal recruiting campaigns have occurred since January 2021, and there have been no participant retention campaigns. However, partially because of the absence of retention campaigns, overall participation has decreased. Modeling performed before the system’s release and on the basis of ED influenza-like illness data before the pandemic indicated AiA would require 5,773 weekly responses to detect the presence of CLI above its baseline rate when SARS-CoV-2 was not in high circulation. Weekly responses have not exceeded 5,773 since June 2023, which initially created concern about the reliability of data after this date. However, correlation with other surveillance metrics remained strong despite the lower number of responses. Existing differences between the survey’s overall respondent population and the LAC population have become more dramatic during the most recent season, which could introduce further bias. The survey also does not account for illnesses among children, and those >60 years of age were underrepresented, which might partially explain its poor correlation with RSV data. Together, those trends indicate that the system’s utility in identifying actionable increases of respiratory illness might be diminished and its representativeness and data quality can be improved.

Among the more consequential differences between AiA and its comparator systems is that AiA was the only system to measure symptomatic illness. This distinction is critical because the informational value of a positive COVID-19 test has changed as testing habits shifted throughout the pandemic, with asymptomatic testing becoming a more common strategy for controlling spread (*21*). However, the undetermined relevance of a positive test in an asymptomatic person made counting positive tests a less reliable metric of determining the community burden of disease. Later, the mass adoption of commercially available antigen tests made it easier for the public to rapidly determine if they had contracted the virus, but those test results were often not reported to public health authorities, creating an environment where case counts were likely underestimates of true disease counts. Pandemic fatigue and high relative costs of testing further contributed to creating uncertainty in the accuracy of raw case counts (*22*–*24*). Although AiA might not have always been able to determine the specific etiology of an illness, AiA’s focus on detecting symptoms enabled it to remain reliable in detecting the magnitude of clinically apparent respiratory illness in the community.

Timely, accurate, and actionable data are crucial in the face of pandemic and seasonal viral respiratory illness. During the COVID-19 pandemic, when typical methods of obtaining data were impaired or inoperable, AiA provided data accurately and in a timely fashion. AiA was able to produce reliable results years into the pandemic despite waning participation and discordant demographic characteristics between respondents and the local population. AiA’s community symptom survey approach enabled it to fill the surveillance gap by gathering data that were not captured by common surveillance methods but were still highly relevant to understanding the community burden of disease. Further, the principles of this survey methodology and the low relative cost of implementation could be adapted in low- and middle-income countries where mobile phone use is widespread but access to healthcare or laboratory testing might be limited. Our findings highlight the strengths of community participatory surveillance systems in monitoring emerging and endemic infectious diseases.

Appendix Additional information about community driven, text message-based COVID-19 surveillance system, Los Angeles County, California, USA, 2020–2024.
