# Supplementary material for: Community-Driven, Text Message–Based COVID-19 Surveillance System, Los Angeles County, California, USA, 2020–2024
Source: Emerg Infect Dis. 2025 Nov;31(11):2109–17. doi: 10.3201/eid3111.250907 (PMC12704538; doi:10.3201/eid3111.250907)
Supplement: Appendix — Additional information about community driven, text message-based COVID-19 surveillance system, Los Angeles County, California, USA, 2020–2024. [file 25-0907-Techapp-s1.pdf]

Article DOI: https://doi.org/10.3201/eid3111.250907

EID cannot ensure accessibility for supplementary materials supplied by authors. Readers who have difficulty accessing supplementary content should contact the authors for assistance.

Community-Driven, Text Message–Based COVID-19 Surveillance System, Los Angeles County, California, USA, 2020–2024

Appendix

Appendix Table. Counts of unique Angelenos in Action respondents, total survey responses, and total COVID-like illness responses by *MMWR* influenza year, Los Angeles County, 2020–2024

Season	Number of respondents	Number of survey responses (Average per respondent)	Number of COVID-like Illness responses (per 1,000 responses)
2019–20*	9,748	89,459 (9.2)	201 (2.2)
2020–21	14,587	473,540 (32.5)	1,170 (2.5)
2021–22	12,242	408,104 (33.3)	2,990 (7.3)
2022–23	9,479	320,872 (33.9)	2,436 (7.6)
2023–24†	7,388	141,903 (19.2)	1,083 (7.6)

*Beginning 2020 *MMWR* week 28.
†Through 2024 *MMWR* week 14.

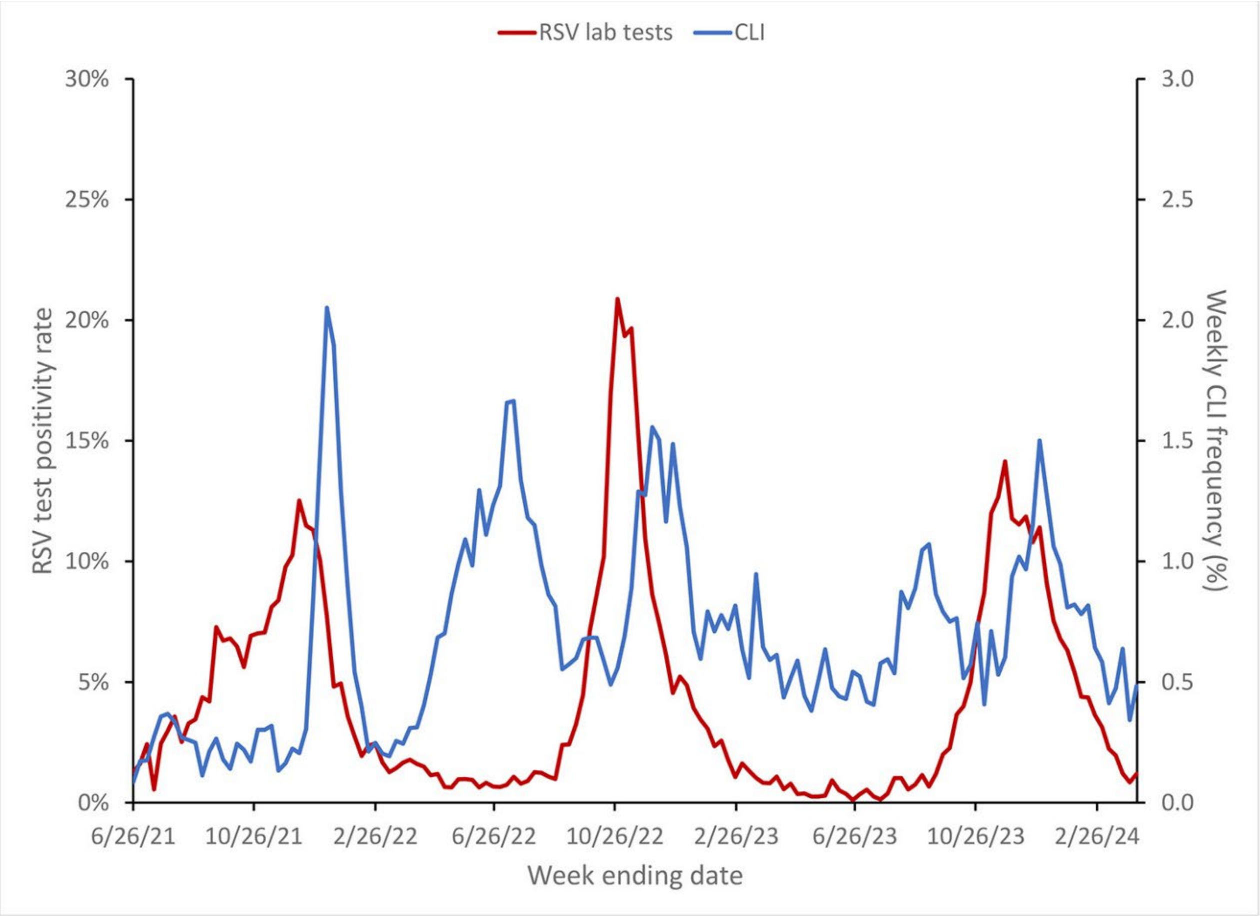

Appendix Figure 1. Trends of weekly COVID-like illness (CLI)* response rate and weekly Los Angeles County sentinel laboratory RSV percent positivity† during June 20, 2021–April 6, 2024.‡ *A response was designated as CLI if the respondent indicated they were sick and answered *yes* to both symptom-specific questions. †Defined as the percentage of tests positive for RSV out of all tests performed for RSV during a given week at the 7 Los Angeles County laboratories with sentinel laboratory designation. ‡Pearson correlation coefficient = 0.094

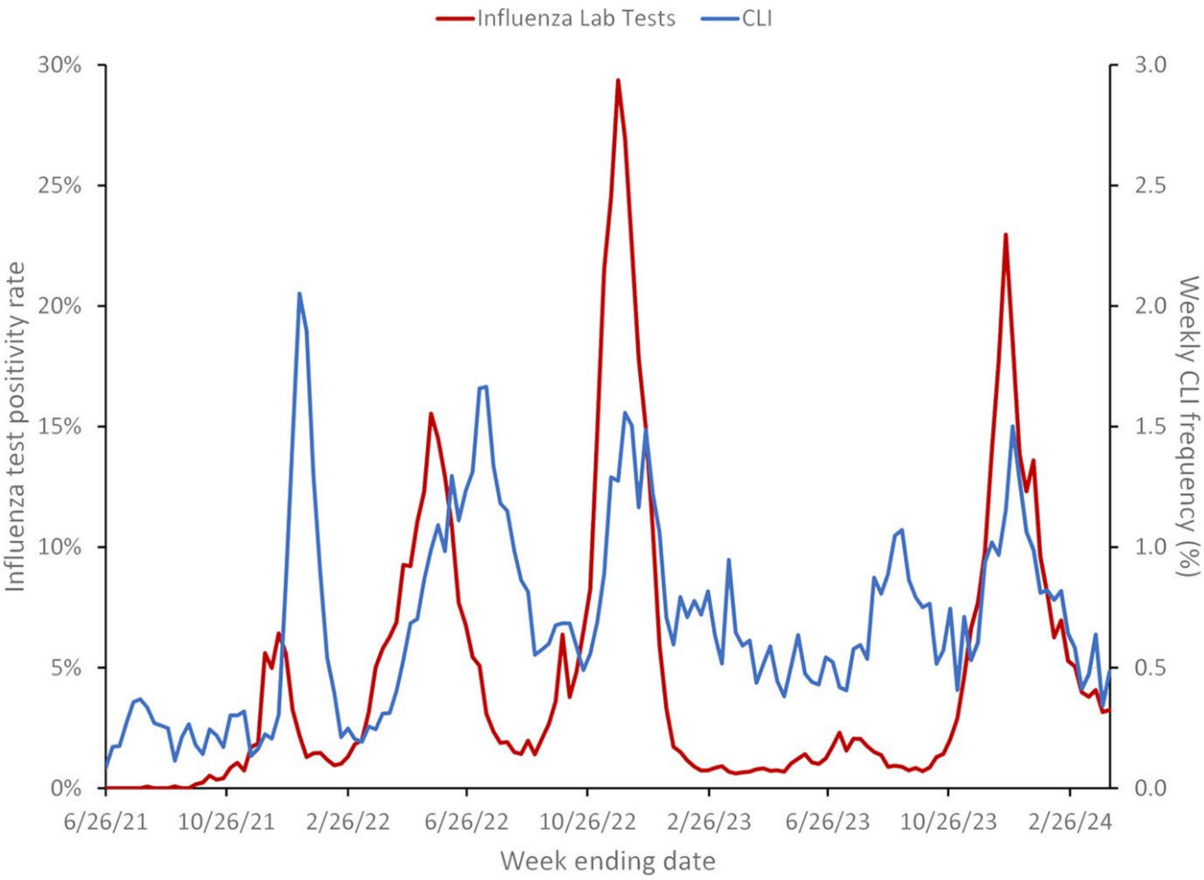

Appendix Figure 2. Trends of weekly COVID-like illness (CLI)* response rate and weekly Los Angeles County sentinel laboratory influenza percent positivity† during June 20, 2021–April 6, 2024.‡ *A response was designated as CLI if the respondent indicated they were sick and answered *yes* to both symptom-specific questions. †Defined as the percentage of tests positive for influenza A or B out of all tests performed for influenza A or B during a given week at the 7 Los Angeles County laboratories with sentinel laboratory designation. ‡Pearson correlation coefficient = 0.508
